# Pro-Angiogenetic Effects of Purified Extracts from *Helix aspersa* during Zebrafish Development

**DOI:** 10.3390/cimb44080232

**Published:** 2022-07-27

**Authors:** Daniela Zizioli, Andrea Mastinu, Alessia Muscò, Sara Anna Bonini, Dario Finazzi, Rosaria Avisani, Giovanni Battista Kron Morelli, Sergio Pecorelli, Maurizio Memo

**Affiliations:** 1 Department of Molecular and Translational Medicine, University of Brescia, viale Europa 11, 25123 Brescia, Italy; daniela.zizioli@unibs.it (D.Z.); alessia.musco@unibs.it (A.M.); sara.bonini@unibs.it (S.A.B.); dario.finazzi@unibs.it (D.F.); sergio.pecorelli@unibs.it (S.P.); 2 ASST degli Spedali Civili di Brescia, Piazzale Spedali Civili, 1, 25123 Brescia, Italy; rosaria.avisani@gmail.com; 3 KMS soc.agr.srl, via Campagnole di sopra 8, 25020 Brescia, Italy; gbkm1948@gmail.com

**Keywords:** zebrafish, embryonic vascular development, angiogenesis, snail derivatives, *Helix aspersa*

## Abstract

*Helix aspersa* is a species of land snail belonging to the Helicidae family, widespread in the Mediterranean and continental area up to Northern Europe. In some areas it is appreciated as a food, but is mostly considered a parasite of gardens and cultivated fields. The mucus of *Helix aspersa* has found multiple applications in the cosmetic and health fields. In the present study, we investigated for the first time the angiogenetic properties of purified extracts from *Helix aspersa* using a transgenic zebrafish line Tg (*kdrl*:EGFP). The angiogenesis induced by purified snail extracts was demonstrated by their capability to increase the three well-established parameters of angiogenesis: generation of intersegmental vessels, modeling of caudal venous plexus, and formation of sub-intestinal venous plexus. The effects appeared to be mediated by the vascular endothelial growth factor (VEGF) pathway, being prevented by pretreatment of embryos with the selective VEGF receptor antagonist SU5416, and supported by the increased *VEGF* mRNA levels found in snail-extract-treated embryos. Insufficient vascular supply is underlined by low VEGF signaling, primarily because of its indispensable role in preventing capillary loss. Our findings might have a pharmacological impact by counteracting VEGF hypofunction and promoting angiogenesis to maintain adequate microvascular and vascular density in normal and suffering tissues and organs.

## 1. Introduction

Today, products of natural origin are of importance from a chemical, analytical, and synthetic point of view, and above all, from a pharmacological point of view [1,2,3,4]. For a long time, land snails have been used as food and for various medical treatments [5]. Snail slime (mucus) is a secretion that favors the adhesive, emollient, moisturizing, lubricating, and defense processes of the snail. Furthermore, this secretion has been used for multiple applications in human medicine and cosmetics [6,7]. Due to their potential application in medicine, the biological effects of extracts from marine and garden snails are now gaining great interest. Several independent studies have shown the ability of snail extracts to act as immunostimulants, and to possess antimicrobial, antifungal, antiviral, and anticancer activities [8,9,10]. Particular attention has been devoted to *H. aspersa*, which is rich in active peptides and glycoproteins mainly present in the mucus [11].

Parallel biochemical and pharmacological preclinical studies have demonstrated that snail mucus exhibits antimicrobial, antioxidant, anti-tyrosinase, and antitumor activities, and that these effects appear to be mediated by different compounds, such as allantoin, hyaluronic acid, peptides, and proteins [12,13,14].

Despite the anecdotal observations of tissue-repairing properties of snail derivatives, their possible effect on angiogenesis has never been investigated. Angiogenesis plays critical roles in vertebrate pathophysiology that range from embryonic development to wound healing and tissue repair [15]. Balance between pro- and anti-angiogenic factors, extracellular matrix components, and endothelial cells is essential for regulating this sophisticated process [16,17]. Blood vessels transport gases, nutrients, waste products, hormones, and circulating cells to all organs of vertebrate organisms. On the other hand, uncontrolled angiogenesis may lead to inadequate changes in tissue vascularization and oxygen availability [16,18]. For example, in humans, insufficient vascular growth contributes to coronary artery disease and critical limb ischemia, while angiogenesis promotes tumor growth [19]. For all of these reasons, understanding and manipulating angiogenic processes can reflect on important therapeutic opportunities.

To elucidate the possible contribution of angiogenesis to the wound healing and tissue repair induced by snail derivatives, we used zebrafish as an in vivo experimental model. The zebrafish (*Danio rerio*) is indeed a useful tool for analyzing developmental processes such as angiogenesis, and compared with other animal models, it offers several advantages. It possesses a closed circulatory system, and the molecular mechanisms underlying its vascular development are highly similar to those of higher vertebrates [20]. Moreover, the embryos are transparent during early development, and can survive through the first week without blood circulation. Much of the signaling controlling angiogenesis appears to be highly conserved among different vertebrate species [21]. In zebrafish, the study of the angiogenesis process usually relies on the investigation of different vasculature structures arising from 22 to 72 h post-fertilization (hpf). In the past, the generation of transgenic zebrafish lines with fluorescent vessels—such as Tg (*kdrl*:EGFP)—allowed real-time observation of vascular growth in vivo, ensuring rapid evaluation of the drug responses of live embryos [22]. Today, the Tg (*kdrl*:EGFP) line represents one of the best in vivo models to visualize the fluorescent intersomitic vessels (ISVs)—the first sign of angiogenesis. They develop around 22 hpf between somites to become part of the trunk vasculature [23]. Another angiogenic process that can be monitored in real time in the Tg (*kdrl*:EGFP) line is the formation and maturation of the caudal venous plexus (CVP)—a network of interconnecting tubules that supports the blood flow in the tail in the early developmental stages. Another important vascular structure that allows observation of the angiogenesis process is represented by the sub-intestinal venous plexus (SIVP). The formation of the SIVP takes place in both the left and right dorsolateral sides of the yolk, starting as early as 30 hpf with migration of cells from the posterior cardinal vein (PCV), and is completed by 72 hpf with the formation of a mature SIV basket connected by intercapillary branches. The SIV basket can be visualized using alkaline phosphatase (AP) staining or whole-mount in situ hybridization (WISH) for endothelial markers [24,25].

The present study aimed to investigate the angiogenic effects of purified extracts from *Helix aspersa* (*H. aspersa*) using zebrafish, and in particular the Tg (*kdrl*:EGFP) line, as an in vivo model. Initially, lyophilizates derived from either the whole snail or its mucus were analyzed by HPLC. Subsequently, the angiogenetic activity was tested from both a morphological and a molecular point of view.

## 2. Materials and Methods

### 2.1. Purified Snail Extracts

The lyophilized of mucus of *Helix aspersa* (LM) and the lyophilized extract of *Helix aspersa* (LH) were supplied by the KMS farm following the protocol shown below. In order to obtain LM, 50 live snails were stimulated with a saturated solution of NaCl by nebulization. Then, the snails were washed, and the secretions were filtered and collected in trays, and subsequently lyophilized. To obtain LH, after 20 min the live snails were frozen at −18 °C, ground, filtered, and lyophilized.

### 2.2. Analytical Procedures

The lyophilized LH and LM were weighed (8 mg) and solubilized in 0.1% trifluoroacetic acid (Merck). Subsequently, they were centrifuged at 10,000× *g* for 10 min, and the supernatant was used for the analyses with the HPLC diode array detector (DAD). A KINETEX 5 µm EVO-C18-100 Å-250 × 4.6 mm chromatographic column with a SHIMADZU LC-2030C HPLC analysis system was used for the analyses [26].

The mobile phases consisted of 0.1% TFA (phase A) and methanol with 0.1% TFA (phase B). The flow was 0.5 mL/min with a pressure below 90 bar. The sample injection volume was 30 µL, and the acquisition wavelength was 200–215 nm.

The most representative components of the LH and LM profiles were isolated, concentrated, and used to evaluate angiogenic tests.

### 2.3. Zebrafish Maintenance and Collection of Eggs

Zebrafish were maintained and used in accordance with the Italian and European rules on animal use. The protocols were approved by the local committee (OPBA) and authorized by the Ministry of Health (Authorization Number 393/2017). For the experiments, two different lines were used: adult AB wild-type zebrafish, and the transgenic line Tg (*kdrl*:EGFP) [27]. The zebrafish lines were raised and maintained in a constant flow of water at 28 °C under a 14 h light/10 h dark cycle, with a pH value of 7.0–7.5 and conductivity between 400 and 500 µs. Fishes were fed with a combination of granular food (from Special Diet Services (SDS), Witham, UK) and freshly prepared Artemia cysts (SDS, Witham, UK). Adult male and female zebrafish were placed in the breeding tanks overnight, and on the next morning freshly spawned eggs could be collected. Unfertilized or dead eggs were removed. Then, these were raised in fish water (0.1 g/L Instant Ocean Sea Salts, 0.1 g/L sodium bicarbonate, 0.19 g/L calcium sulfate) with incubation at 28 °C until the experiments. The staging of zebrafish embryos was carried out as described by Kimmel et al. [28]. Embryos for WISH and AP were treated with 0.003% 1-phenyl-2-thiourea (PTU) (Sigma-Aldrich, St. Louis, MO, USA) to prevent pigmentation.

### 2.4. Treatment with Purified Snail Extracts

Fresh solutions of purified snail extracts were prepared on the day of the experiment in fish water/0.1% DMSO, and then zebrafish embryos were treated at 4 hpf and incubated at 28 °C. Exposure of purified snail extracts to zebrafish embryos was performed via immersion method—embryos at the desired stage were placed in a 100 mm × 15 mm Petri dish containing either snail solution (treated) or the vehicle (control), and incubated under static conditions (i.e., no daily solution renewal).

Fish water with 0.1% DMSO was used as a negative control (embryo mortality = 10%), while 3,4-dichloroaniline (3.7 mg/L) was used as a positive control [29].

Mortality of controls and treated embryos was recorded at 48 hpf. The optimal dose did not induce lethality in zebrafish embryos exposed from 4 to 48 hpf. The survival rate of each compound was calculated, and a dose–response survival graph was plotted at 48 hpf using GraphPad8. We selected 10 µg/mL as the optimal dose of purified snail extracts.

**Figure 1:**
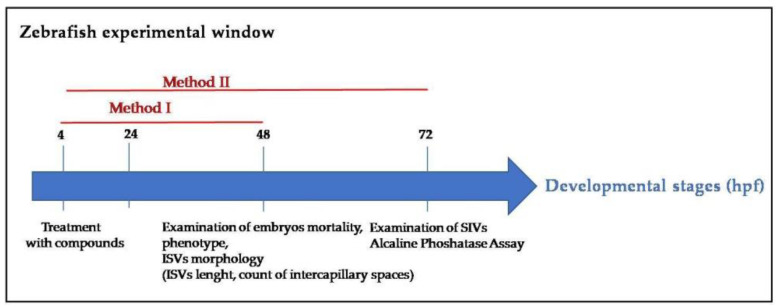
Exposure methods used as experimental plans: The transgenic Tg (*kdrl*;EGFP) zebrafish embryos were treated with the optimal dose (10 µg/mL) of snail extracts (LH, LM, LH3, and LM2) from 4 to 48 hpf (Method I) or from 4 to 72 hpf (Method II). The embryos treated via Method I were used to establish the dose curve and the mortality rate. Embryos treated with optimal doses via Method I were subjected to the analysis.

We designed an experimental plan as follows: we first treated the transgenic Tg (*kdrl*-EGFP) embryos via two exposure methods (see Materials and Methods) to examine their effects on the ISV and CVP (Method I, 4–48 hpf) or SIV development (Method II, 4–72 hpf) (Figure 1).

### 2.5. VEGF Inhibitor Treatment

Zebrafish embryos were then treated at 4 hpf with the snail derivatives LH, LM, LH3, and LM2, and then at 15 hpf with the VEGF inhibitor SU5416 (Sunitinib, Merck KGaA, Darmstadt, Germany) at a final concentration of 5 µg/mL in fish water and incubated in 3 cm diameter well-plates. Treated and untreated embryos were incubated at 28 °C and staged at 36 hpf for WISH.

### 2.6. Confocal Microscope Imaging

Vehicle- and snail-extract-treated Tg (*kdrl*:EGFP) zebrafish embryos were embedded in agarose and placed in a small chamber on a suitable support for subsequent microscopic analysis. Zebrafish were examined using a Zeiss LSM 510 META confocal laser scanning microscope (Carl Zeiss, Jena, Germany), using an objective Achroplan 10×/0.25 and a 488 nm laser. Three-dimensional images of zebrafish vessels were performed using the ZEN black software on z-stack scan images (15-section stacks, 8 µm interval sections).

### 2.7. Image Analysis of ISVs and CVPs

Measurement of the length and width of ISVs was performed in Tg (*kdrl*:EGFP) embryos treated or not treated with purified extracts from *H. aspersa*. Dead embryos and embryos exhibiting target effects of toxicity, such as pericardial edema, were excluded from the experiments. At 48 hpf, fluorescent images depicting the vascular tree were acquired for the control and treated groups. The images were taken with a confocal microscope.

The images were then processed, and the length and width of the ISVs was measured with ImageJ Fiji software 1.8.0 (Rasband, W.S., ImageJ, U. S. National Institutes of Health, Rockville, Bethesda, MD, USA). Two landmarks—one dorsal and one ventral—were established to define the section of ISVs to be measured: ventrally, the commencement of the ISV at the upper border of dorsal aorta; dorsally, the bifurcation of ISVs into dorsal longitudinal anastomotic vessels (DLAVs). To measure the length of the ISVs, 10 embryos per group (3 different groups) were analyzed. For each embryo, 4 intersegmental vessels corresponding to the yolk sac extension were selected. The width of the ISVs was determined according to the quantification method described by Hans et al. [30].

A region of interest (ROI) enclosing 4 ISVs corresponding to the yolk sac extension was selected for each embryo. The ROI was then converted to greyscale and thresholded to obtain the bidimensional tracing of ISVs over a background hull. The width of each ISV was determined as normalized ISV area, calculated by dividing the area of each ISV trace by the area given by the background. The same images were also utilized to manually count the numbers of intercapillary spaces in the CVPs of control and treated embryos, following the protocol described in [31,32].

### 2.8. Whole-Mount In Situ Hybridization (WISH)

Wild-type zebrafish embryos were raised with or without purified snail extracts from *H. aspersa*. At 26 hpf, 20 embryos without any obvious deformities were selected for each condition and fixed with 4% (*v*/*v*) paraformaldehyde (PFA) (Sigma-Aldrich, St. Louis, MO, USA), dehydrated in 100% (*v*/*v*) methanol, and stored at −20 °C. WISH was performed with the vascular probe *fli1.* The preparation of the probes and WISH in zebrafish embryos was performed as previously described in [33]. WISH images were taken with a Leica MZ16F stereomicroscope equipped with a DFC 480 digital camera and LAS Leica Imaging software (Leica, Wetzlar, Germany) at 63× magnification.

WISH images were quantified using ImageJ Fiji software, as follows: The region of the embryo tail containing the WISH signal (approximately from the mid-yolk region to the tip of the tail) was selected, and the intensity was measured. An equal area of the tail outside of the stained area was selected to determine the background. The value of the colorimetric signal was then obtained by subtracting the background from the measured intensity.

### 2.9. AP Staining

AP assay was performed to visualize the ectopic sprout formation of the SIVP in zebrafish embryos after treatment with purified snail extracts. At 4 hpf, wild-type (AB) zebrafish embryos (*n* = 20) (in duplicate) were treated with LH, LM, LH3, and LM2 at 10 µg/mL, and embryos treated with fish water/0.1% DMSO were used as controls. At 24 hpf, 0.003% PTU was added to the embryos to prevent pigmentation. The embryos were evaluated for any mortality and toxicity every 24 h. Any dead embryos or those showing toxic effects (i.e., abnormal phenotype) were removed. Embryos with pericardial edema and tail abnormalities (e.g., hyperextension, flexion, lateral bending) were considered to be of an abnormal phenotype. At 72 hpf, embryos from each condition and the control group were fixed in 4% (*v*/*v*) PFA. The AP assay was performed as described elsewhere [34]. Briefly, embryos were put in 100% (*v*/*v*) methanol–phosphate-buffered saline (PBS) solution. The embryos were then equilibrated in Tris buffer (100 mM Tris-HCl (pH 9.5), 50 mM MgCl₂, 100 mM NaCl, 0.1% Tween-20) and stained with nitro blue tetrazolium chloride (NBT) and 5-bromo-4-chloro-3′-indolyphosphate *p*-toluidine salt (BCIP) solution. The stained SIVP was evaluated under a Zeiss Axiozoom V13 (Zeiss, Jena, Germany) microscope at 60× magnification. In particular, the ICVs in the SIVP were manually counted in all experimental groups [24].

The images were taken in lateral position at 32× magnification with a Zeiss Axiozoom V13 (Zeiss, Jena, Germany) microscope, equipped with a PlanNeoFluar Z 1×/0.25 FWD 56 mm lens and Zen Pro software.

### 2.10. RNA Extraction, Reverse Transcription, and RT-qPCR

Total RNA extraction was performed with the guanidine isothyocianate/phenol method. Frozen pooled embryos (30 embryos/sample) were lysed in TRI reagent^®^ (Sigma-Aldrich, St. Louis, MO, USA) according to the manufacturer’s protocol. RNA was isolated using 1-bromo-3-chloropropane (Sigma-Aldrich), precipitated in isopropanol (Sigma-Aldrich), and resuspended in RNase-free water (Thermo Fisher Scientific, Waltham, MA, USA). Quantification was performed with a mySPEC micro-volume spectrophotometer (VWR International, Philadelphia, PA, USA). One microgram of RNA was retrotranscribed using the QuantiTect Reverse Transcription Kit (QIAGEN, Hilden, Germany). RT-qPCR was performed using the Viia7 system (Thermo Fisher Scientific, Waltham, MA, USA). Reactions were performed in 10 µL volume, with 2 µM of each primer, 10 µL of SYBR Green Master Mix (Bio-Rad, Hercules, CA, USA), and 20 ng of cDNA. The amplification profile consisted of a 1 min initial denaturation step at 95 °C, followed by 40 cycles of two-step amplification (95 °C for 15 s and 60 °C for 30 s) and a melting cycle. Each reaction was performed in triplicate, and the relative expression of each gene was calculated with the ΔΔCt method, using rpl13a as a reference gene. The primer sequences are listed in Table 1.

### 2.11. Statistical Analysis

The data were collected for the control group and the treated groups. Each experiment was repeated a minimum of two or three times. The data are presented as the mean ± SEM. All graphs were plotted using GraphPad Prism 8. The significance of all of the data was analyzed by one-way analysis of variance (one-way ANOVA); *p*-values less than 0.05 were considered to be significant; * *p* < 0.05, ** *p* < 0.005, *** *p* < 0.0005.

## 3. Results and Discussion

### 3.1. Analytical Profile of Purified Extracts from Helix aspersa

**Figure 2:**
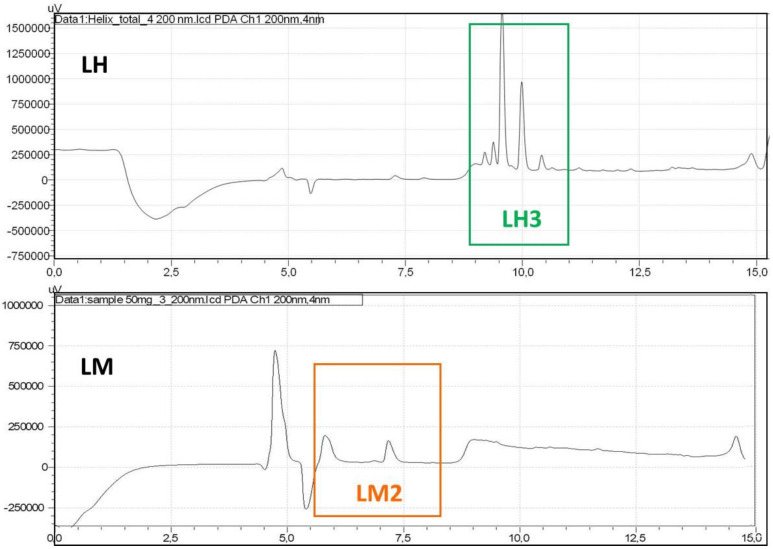
(**Top**) HPLC chromatograms of lyophilized extracts of *H. aspersa* (LH) and (**Bottom**) mucus of *H. aspersa* (LM) solubilized in 0.1% TFA (trifluoroacetic acid) and obtained at 200 nm.

Lyophilized extracts from *H. aspersa* (LH) and the mucus of *H. aspersa* (LM) were analyzed by HPLC. The LH representative chromatogram in Figure 2 (top) shows the presence of five main components in the elution range between 8.5 and 11.5 min. These fractions were isolated and referred to as LH3. The chromatogram of the lyophilized mucus (LM) shows two main components in the elution intervals around 6 and 7.5 min (Figure 2 (bottom)). These fractions were collected and referred to as LM2.

All extracts (LH, LH3, LM, and LM2) were concentrated and tested for angiogenesis in zebrafish during embryonic development.

### 3.2. Purified Extracts from Helix aspersa Induced Pro-Angiogenetic Effects during Zebrafish Development

The study on the effects of purified extracts from *H. aspersa* during angiogenesis was focused on three well-established parameters of angiogenesis: development of ISVs, modeling of the CVP, and formation of the SIVP.

Preliminary studies were carried out to define the optimal concentration for each purified extract to be used in the next experiments. Embryos were exposed to the purified snail derivatives LH, LM, LH3, and LM2 at the following concentrations: 5, 10, 30, 50, and 100 µg/mL. Control embryos were exposed to fish water plus 0.1% DMSO. After 48 hpf, the embryos were analyzed for toxicity and morphological changes (Figure S1). All compounds tested at the concentration of 10 µg/mL were found to be non-toxic and well tolerated by the embryos. This concentration was used for the following experiments.

#### 3.2.1. ISV Formation

During the embryonic development of zebrafish, angiogenesis is best characterized by the formation of ISVs and the SIVP. Vascular development initiates at around 12 hpf, when hemangioblasts first appear along the lateral plate mesoderm. At 24 hpf, two axial vessels—the dorsal aorta (DA) and posterior cardinal vein (PCV)—are first formed in the trunks of the embryos. Meanwhile, bilateral ISVs sprout dorsally from the DA, and finally reach the dorsal-most region of the somites and split dorsolaterally to form the dorsal longitudinal anastomotic vessels (DLAVs). The ISVs, originating from the common cardinal vein (duct of Cuvier) at 48 hpf, form in the shape of a basket on the dorsolateral surface of the yolk at 3 dpf [23]. To examine the effects of purified snail derivatives on vascular development, we first used the transgenic zebrafish line Tg (*kdrl*:EGFP), which presents a vascular-endothelium-specific *kdrl* promoter directing EGFP expression. Kdrl is a kinase insert domain receptor-like protein also known as vascular endothelial growth factor receptor 2 (VEGFR-2) [35]. In the presence of pro-angiogenic signals, the morphology of nascent fluorescent vessels can be easily observed, and ISVs can be measured [36]. In addition, increased angiogenesis can be monitored by following the formation of intercapillary spaces in the CVP. The CVP forms a dense capillary network in the tails of the embryos, and the presence of the intercapillary spaces indicates a proper remodeling of blood vessels [31].

**Figure 3:**
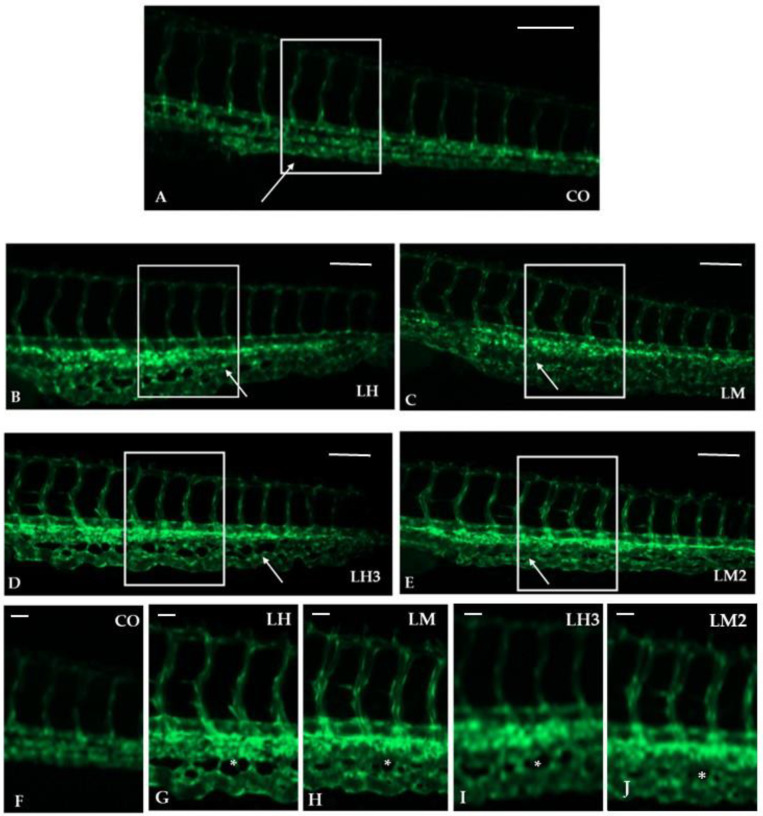
Purified extracts from *H. aspersa* caused pro-angiogenetic effects on ISVs and intercapillary spaces in the CVP: (**A**–**E**) Representative images of zebrafish trunk ISVs of controls (fish water and 0.1% DMSO) (**A**) and treatments with 10 µg/mL of LH (**B**), LM (**C**), LH3 (**D**), and LM2 (**E**). All figures are lateral views with dorsal to the top and anterior to the left, and fluorescent vessels of embryos were observed at 48 hpf. White arrowheads indicate the CVP, showing an increase in the angiogenesis process. Enlarged white boxes indicate the magnification of ISVs and CVPs (**F**–**J**). Asterisks (*) indicate the intercapillary spaces. The experiments were repeated twice. Images were taken with a Zeiss LSM 510 META confocal laser scanning microscope (Carl Zeiss, Jena, Germany), using an objective Achroplan 10× and 20×/0.25 and a 488 nm laser, and represent 1 embryo out of 30 with the same phenotype (for more details, see Section 2).

**Figure 4:**
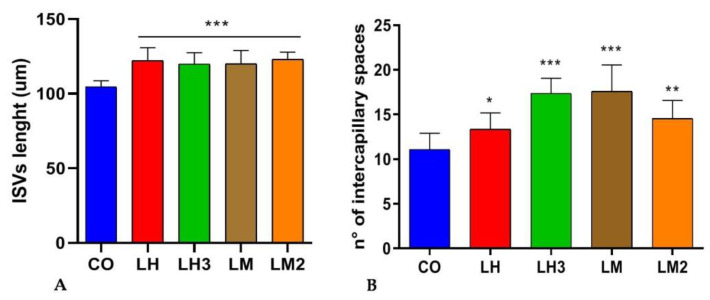
The graphs represent the mean values obtained for ISV length (**A**) and the average numbers of intercapillary spaces in control and treated embryos (**B**). Data are representative of two replicates, and shown as the mean ± standard deviation; *** *p* < 0.001 vs. control group; ** *p* < 0.005 vs. control group; * *p* < 0.05 vs. control group; *n* = 15 from two independent experiments. Statistical analysis was performed with GraphPad version 8.3.3 (GraphPad Software, Inc., La Jolla, CA, USA).

As shown in the representative images in Figure 3, treatment of zebrafish embryos with LH, LM, LH3, and LM2 caused a strong upregulation of the angiogenesis process. The length of ISVs in embryos exposed to the different snail extracts was found to be significantly increased in comparison to controls (Figure 4). These results suggest that the purified snail derivatives had effects on the initial development of the trunk vasculature. In the treated embryos, the CVP formed as a very dense capillary network with several spaces between capillaries, as compared to controls (Figure 3). We counted the number of intercapillary spaces in the CVP as described by Shoam et al. [31], and found them to be increased from 10 ± 0.9 (control) to 13 ± 0.97, 17 ± 0.82, 17 ± 0.96, and 14 ± 0.92 in the embryos exposed to fish water plus LH, LH3, LM, and LM2, respectively (Figure 4).

#### 3.2.2. SIVP Formation

To further investigate the effects of LH, LM, LH3, and LM2 during the angiogenesis process, we monitored the SIVP’s development by staining for endogenous alkaline phosphatase activity at 72 hpf. In the past, several authors demonstrated that the SIVP can be used as an easily visible vascular network to screen molecules with angiogenetic properties [37,38]. The sub-intestinal venous plexus is a bilateral vascular structure derived from duct of Cuvier, and is directly in contact with the yolk syncytial layer (YSL). Therefore, the SIVP is likely a network of vessels that mediates the uptake and circulation of nutrients from yolk to embryo, and shows different sensitivity to the most popular growth factors [23,24].

**Figure 5:**
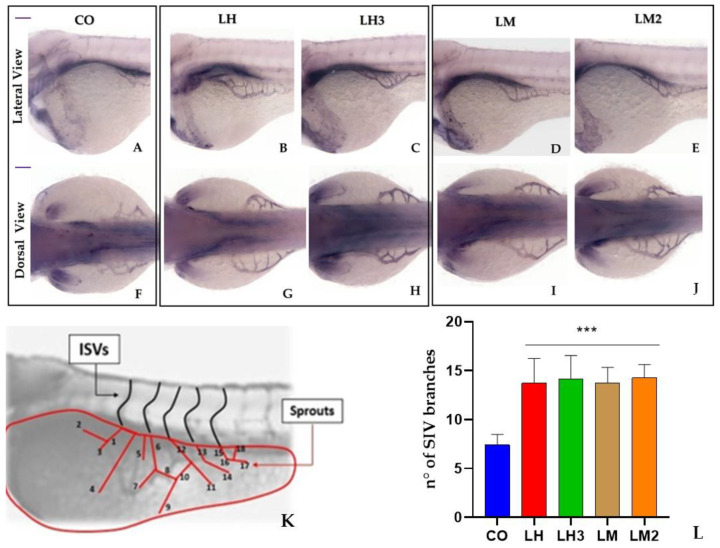
Purified extracts from *Helix aspersa* caused an increased number of interconnecting vessels in the SIVP: Images from (**A**) to (**J**) show representative staining of an AP assay performed in control and treated embryos, with enlargement of the SIVP region at 72 hpf. (**A**–**E**) lateral view; (**F**–**J**) dorsal view. The scheme in (**K**) depicts the counting of ectopic sprouts. The graph in (**L**) shows the average number of SIVP branches. Data are representative of two replicates (*n* = 20 for each group) and are shown as the mean ± standard deviation; *** *p* < 0.001 vs. control group. Statistical analysis was performed with GraphPad version 8.3.3 (GraphPad Software, Inc., La Jolla, CA, USA).

Zebrafish embryos were treated with purified snail extracts or 0.1% DMSO, and the interconnecting branches were counted following the protocol described by Basnet et al. (2021) [36]. Snail extracts modified the developmental patterning of the SIVP, as evidenced by the additional branches and the ectopic sprouting vessels outside the SIVP basket. This allowed us to infer that different snail derivatives can exert pro-angiogenic activity in zebrafish embryos. The quantification of this effect on the SIVP was measured by counting the number of the branches; snail derivatives increased the number of branches by 97% when compared to the control group (Figure 5). This result indicates that purified snail extracts induce neovascularization in zebrafish SIVPs.

### 3.3. Pro-Angiogenic Effects of Snail Derivatives

We were than interested in exploring the molecular mechanisms underlying the pro-angiogenetic effects of purified extracts from *H. aspersa*. Extrinsic cues and intrinsic receptors guide the morphogenesis of the vascular system. First, we measured mRNA levels of *vegfa*, which is one of the main growth factors involved in angiogenesis. Numerous signaling pathways involving Vegf, Notch, and chemokines are known to ensure proper angiogenic growth of the complex vascular network in the developing zebrafish embryos [20,39]. Vegf and its tyrosine kinase receptors (Vegfr) are key regulators of angiogenesis and are highly conserved across vertebrate species. Vegfa is the most important member of the Vegf family. Vegfa binds to two related endothelial-cell-specific receptors: Vegfr-1 (*flt1*) and Vegfr-2 (*kdrflk1*) [40]. In zebrafish, Vegfa is secreted by somitic tissue to induce sprouting and growth of the intersegmental vessels from the dorsal aorta, along with ISV formation [41,42], and its expression is induced by sonic hedgehog (shh) expression at the midline [43].

**Figure 6:**
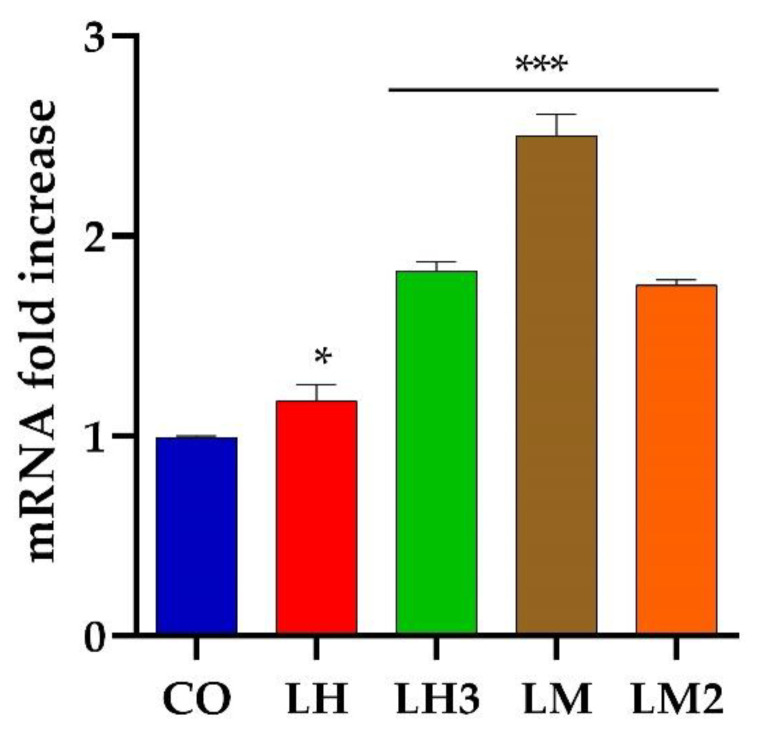
Expression level analysis of *vegfa* as an angiogenesis marker: Embryos were treated with purified extracts from *H. aspersa* (LH, LM, LH3, and LM2) and analyzed at 48 hpf. Gene expression was normalized using *rpl13a* as a reference gene and expressed as the mRNA fold increase. Data are representative of three replicates, and are shown as the mean ± standard deviation; *** *p* < 0.001 vs. control group; * *p* < 0.05 vs. control group. Statistical analysis was performed with GraphPad version 8.3.3 (GraphPad Software, Inc., La Jolla, CA, USA).

As shown in Figure 6, *vegfa* mRNA expression levels increased in zebrafish embryos exposed to 10 µg/mL LH, LM, LH3, or LM2. In this regard, LM was the most active fraction, inducing a 2.5-fold increase.

**Figure 7:**
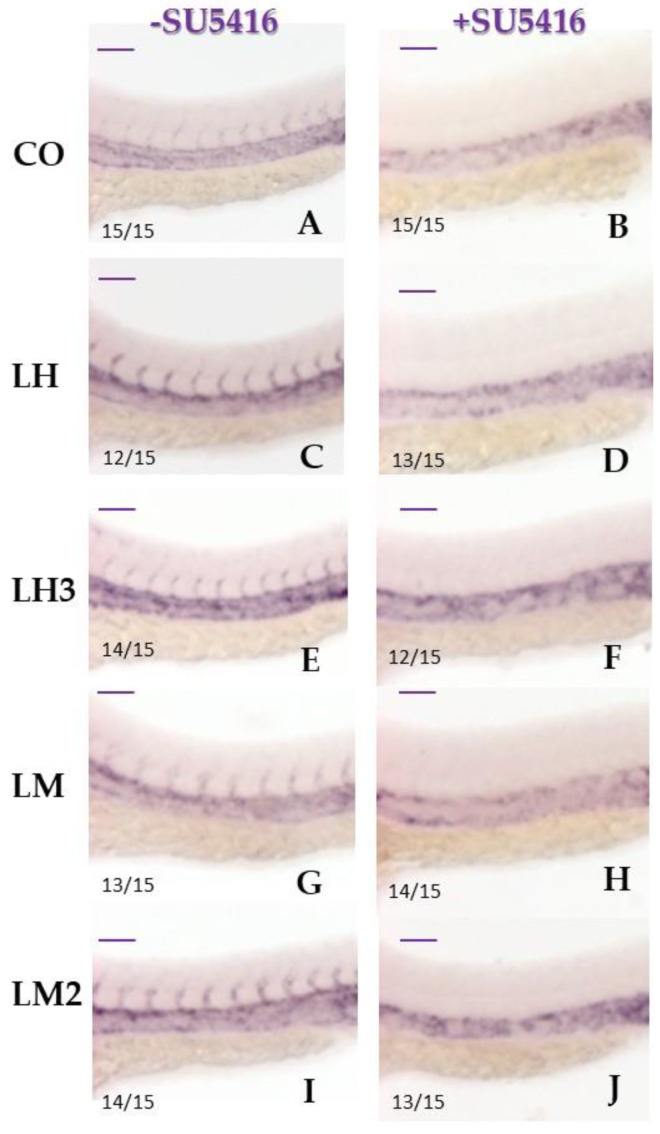
Pretreatment with a VEGF pathway inhibitor prevented the formation of intersomitic vessels: The panels show representative images of a WISH assay performed with *fli1* as an endothelial marker, at 36 hpf, in untreated (**A**,**C**,**E**,**G**,**I**) and treated embryos (**B**,**D**,**F**,**H**,**J**) with SUGEN 5416. Previously, some embryos were treated with snail derivatives via Method I and controls with fish water and 0.1% DMSO (see Materials and Methods, Section 2.4 and Section 2.5). Magnification of the trunk region (32×). Two replicates were performed (*n* = 15). Ratios at the bottom-left part of each picture specify the number of embryos showing the same staining pattern, compared to the total number of embryos used for each experiment. The images were taken in lateral position at 32× magnification with a Zeiss Axiozoom V13 (Zeiss, Jena, Germany) microscope, equipped with a PlanNeoFluar Z 1×/0.25 FWD 56 mm lens and Zen Pro software.

Next, we studied the expression levels of the endothelial vascular marker *fli1* by in situ hybridization in embryos pretreated with the VEGF receptor antagonist SU5416. This molecule is a selective inhibitor of the Flk-1/KDR receptor tyrosine kinase, known to block VEGF-induced vascular endothelial cell proliferation in the angiogenesis process [44]. The *fli1* gene belongs to the ETS family; it is expressed at high levels in endothelial cells, and regulates the angiogenesis process by controlling the expression of key angiogenic genes [45]. Pretreatment with SU5416 completely prevented the formation of ISVs in the dorsal aorta and the caudal vein in both control and treated embryos (Figure 7). These results suggest that the VEGF pathway might play a role in the pro-angiogenic effects of purified snail extracts.

## 4. Conclusions

In the present study, we demonstrated an additional biological activity of purified *H. aspersa* extracts, possibly related to their tissue-repair potential: promotion of angiogenesis. Indeed, the treatment of zebrafish embryos with the extracts from 4 to 48–72 hpf induced the generation of intersegmental vessels, modeling of the caudal venous plexus, and the formation of the sub-intestinal venous plexus. Zebrafish embryos are a very useful experimental model for investigating the molecular and cellular mechanisms controlling the formation of different vasculature structures arising during development.

The pro-angiogenic effects of *H. aspersa* appeared to be mediated by the Vegf pathway; indeed, a pretreatment with the selective vegf receptor antagonist SU5416 prevented the formation of intersomitic vessels; furthermore, snail-extract-treated embryos presented increased *vegfa* mRNA levels. These results suggest that *H. aspersa* itself and the mucus it secretes contain substances capable of increasing Vegf expression and, in turn, promoting angiogenesis. We are not yet able to identify the molecule(s) responsible for such effects. Based on the analytical data, they are hydrophilic, polar in nature, and present in both *H. aspersa* snails and their mucus.

To our knowledge, this is the first in vivo demonstration of the angiogenic properties of purified snail derivatives. These findings might have a pharmacological impact. Vascular endothelial growth factor (Vegf) is the angiogenic factor promoting and orchestrating most—if not all—processes of neovascularization that take place in the embryos and adults of different animal models. Vegf is also required to sustain newly formed vessels and plays multiple additional roles in the maintenance and function of certain mature vascular beds. Because different vascular networks display highly variable dependencies on Vegf for survival, perturbations in Vegf signaling may impact organ homeostasis in multiple ways. Indeed, as in other animals, the potential consequences of Vegf loss of function or hypofunction may trigger the regression of many Vegf-dependent vasculatures, such as in aging or common human lung pathologies, including emphysema and respiratory distress syndrome, as previously reported in [46,47]. Counteracting Vegf hypofunction and promoting angiogenesis might maintain adequate microvascular density, allowing the provision of oxygen and blood-borne substances to suffering tissues and organs.

It will be interesting, as a future step of this study, to test the use of these extracts on a zebrafish model of pathology, inducing a situation of tissue damage (e.g., ischemic damage) for potential use of the extracts in a therapeutic context. Before proceeding with future developments, however, these results should be further supported by confirmation of the molecular mechanism(s) involved; indeed, the Vegf results will be further investigated using sequencing approaches.

## Figures and Tables

**Table 1:** Primer sequences.

Gene	Forward	Reverse
*vegfa*	CTCCATCTGTCTGCTGTAAAGG	GGGATACTCCTGGATGATGTCTA
*Dre rpl13a*	TCTGGAGGACTGTAAGAGGTATGC	AGACGCACAATCTTGAGAGCAG

## Data Availability

Data are available from the corresponding author upon request.

## Supplementary Materials

The following are available online at https://www.mdpi.com/article/10.3390/cimb44080232/s1, Figure S1: Dose curve at 48 hpf.
