# Supplementary figures and images for: Pro-Angiogenetic Effects of Purified Extracts from Helix aspersa during Zebrafish Development
Source: Curr Issues Mol Biol. 2022 Jul 27;44(8):3364–77. doi: 10.3390/cimb44080232 (PMC9406997; doi:10.3390/cimb44080232)

Figure S1

Dose curve 48 hpf

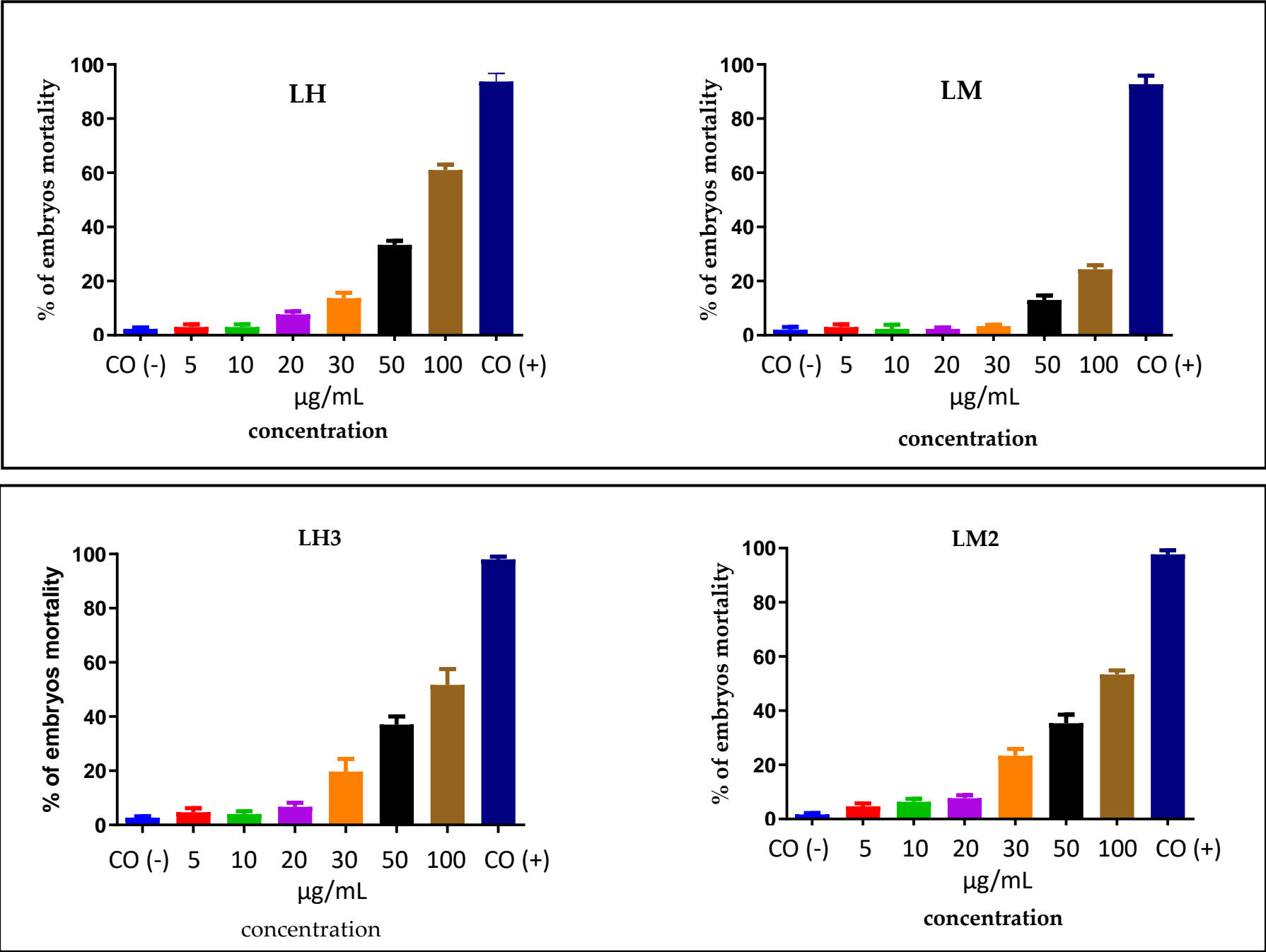

Supplement: Supplementary file 1 [file cimb-44-00232-s001.zip › cimb-1802644-supplementary.pdf]
